# Systematic review of predictors of hospitalisation for non-specific low back pain with or without referred leg pain

**DOI:** 10.1371/journal.pone.0292648

**Published:** 2023-10-10

**Authors:** Joseph F. Orlando, Matthew Beard, Michelle Guerin, Saravana Kumar

**Affiliations:** 1 UniSA Allied Health & Human Performance, University of South Australia, Adelaide, Australia; 2 Central Adelaide Local Health Network, Adelaide, Australia; UNESP: Universidade Estadual Paulista Julio de Mesquita Filho, BRAZIL

## Abstract

Significant costs and utilisation of healthcare resources are associated with hospitalisations for non-specific low back pain despite clinical guidelines recommending community-based care. The aim of this systematic review was to investigate the predictors of hospitalisation for low back pain. A protocol was registered with PROSPERO international prospective register of systematic reviews (#CRD42021281827) and conducted in line with the Preferred Reporting Items for Systematic Reviews and Meta-Analyses (PRISMA) 2020 statement. Database search of Ovid Medline, Emcare, Embase, PsycINFO, Cochrane Library, PEDro and OTSeeker was conducted. Studies were included if they examined a predictor of hospitalisation for non-specific low back pain with or without referred leg pain. Data was extracted and descriptively synthesised. Risk of bias of included studies was assessed using the Critical Appraisal Skills Programme Checklists. There were 23 studies published over 29 articles which identified 52 predictor variables of hospitalisation for low back pain. The risk of hospitalisation was grouped into themes: personal, health and lifestyle, psychology, socioeconomic, occupational, clinical, and health systems and processes. There was moderate level evidence that arrival to an emergency department via ambulance with low back pain, and older age increase the risk of hospitalisations for low back pain. There was low level evidence that high pain intensity, past history of low back pain, opioid use, and occupation type increase the risk of hospitalisation for low back pain. Further research into psychological and social factors is warranted given the paucity of available studies. Hospital avoidance strategies, improved patient screening and resource utilisation in emergency departments are considerations for practice.

## Introduction

Low back pain (LBP) is a highly prevalent condition and leading cause of disability [1, 2]. It represents the 9^th^ most common condition in general practice [3] and 4.4% of all emergency department presentations worldwide [4]. Most cases of LBP represent non-serious spinal pathology [5] with clinical guidelines supporting active self-management in community-based care [6, 7].

There are challenges with implementation of evidence-based practice for LBP relating to both clinician and patient factors [8, 9]. Management of LBP that diverges from best practice contributes to higher rates of spinal imaging [10–12], opioid prescriptions [12, 13] and specialist referrals [12]. This can lead to poorer clinical outcomes and risk of harm to patients [14, 15] with increased healthcare and societal costs [16].

The burden and costs of LBP are substantial when seeking hospital-based care. A large multicentre observational study calculated a mean cost of AUD$13,137 per hospitalisation for LBP with an average length of stay of nine nights [17]. The reasons for people being hospitalised for LBP are complex and multifactorial, but emerging evidence indicates that this patent group presents with high pain severity, functional impairment and helplessness [18–20]. Little is known about the risk factors of hospitalisation for LBP and no systematic reviews have been identified on this topic. Understanding the risk factors for hospitalisation may reveal insights into better supporting people who present to hospital with LBP to manage in community-based care whilst reducing the burden and costs on hospital systems. This study aims to address the knowledge gap in understanding the predictors of hospitalisation in people with LBP with or without referred leg pain.

## Materials and methods

### Protocol and registration

A protocol was registered with PROSPERO international prospective register of systematic reviews (#CRD42021281827). This review was conducted and reported in line with the Preferred Reporting Items for Systematic Reviews and Meta-Analyses (PRISMA) 2020 statement [21]. Refer to S1 Checklist.

### Search strategy

Seven electronic databases were searched, including: Ovid Medline, Emcare, Embase, PsycINFO, Cochrane Library, PEDro and OTSeeker. The following search terms were used with MESH headings: exp back pain, exp low back pain, exp backache, exp sciatica and exp hospitalisation. The search was limited to humans, English language and published from inception to 1^st^ November 2022. Grey literature searching was undertaken using a commonly available internet search engine (Google Scholar) and searching of reference lists of included studies was performed to identify additional records.

All search results were pooled and duplicates removed. Titles and abstracts were screened before analysing the full texts to determine their eligibility. The screening process was undertaken by two independent reviewers (JO, SK). Any disagreements were resolved and discussed with a third reviewer (MG), where required.

### Study selection

All forms of primary research were considered, including prospective or retrospective cohort studies. Secondary research, such as literature reviews were excluded but their reference lists were searched to identify additional records. Studies were included if they examined a predictor of hospitalisation for non-specific LBP with or without referred leg pain. Hospitalisation was defined as a minimum overnight stay as a hospital inpatient. Predictors of hospitalisation expressed as risk ratio, odds ratio or equivalent were included in the review. There was no exclusion on the time at which data was captured, either before, during or following the LBP episode. All clinical contexts were considered, including emergency departments, inpatients, outpatients and community health clinics. Participants had to be adults 18 years of age or older at the time of hospitalisation for non-specific LBP including sprains and strains of the lumbar spine (disc, joints, muscles, ligaments) or sciatica/ radicular leg pain. Studies that included participants with specific spinal disorders (fracture, cauda equina syndrome, myelopathy, neoplasm, infection, axial spondyloarthropathy, radiculopathy or claudication with neurological loss, deformity) were included provided that data for non-specific LBP was presented separately from specific spinal disorders and that the latter represented less than 5% of participants in studies set in primary care [5] or less than 10% of participants in studies set in tertiary care [22], as is reflected in clinical practice. Exclusion criteria included participants who were children at the time of hospitalisation; primary complaint of neck or thoracic spinal pain; back pain secondary to non-spinal disorders (visceral, vascular, urogenital, pelvis/hip, widespread pain disorder); back pain secondary to pregnancy; or hospitalisation for elective spinal surgery. Studies that included data on hospitalisations for medical and surgical management of LBP were included, provided the former was presented separately and the latter could be excluded from this review. Refer to PICOTS table in S1 Appendix.

### Risk of bias assessment

The Critical Appraisal Skills Programme (CASP) was used to assess the risk of bias of included articles based on the study design: CASP Cohort Study Checklist [23] or CASP Case Control Study Checklist [24]. Items included: study purpose, recruitment, exposure and/or outcome measurement, confounders, follow-up, results, statistical precision, believability, generalisability, consistency with other research, and practice implications. The individual components were rated as ‘yes’, ‘no’, ‘unclear’ or ‘not applicable’. Some items were ‘not applicable’ for rating as they comprised descriptive responses. Articles’ risk of bias was then rated as low, medium or high and used to discuss the overall findings. Articles were not excluded based on the risk of bias. The risk of bias assessment of included articles was undertaken by two independent reviewers (JO, SK). Any disagreements were resolved and discussed with a third reviewer (MG), where required.

### Data extraction, synthesis and analysis

The data were extracted and collated into bespoke Microsoft Excel® spreadsheets by one reviewer (JO) and a sample cross-checked by a second reviewer (SK). The following domains were used to collate data: study design, level of evidence, setting, sample size, participant details (age, gender), data sources, data capture period and follow-up, diagnostic criteria, hospitalisation details (length of stay, percentage hospitalised), predictor variables and findings. The predictor variables from each article were expressed as its odd ratio (OR), risk ratio (RR), hazard ratio (HR), prevalence ratio (PR), or equivalent and tabulated for analysis. Meta-analysis was initially considered, but preliminary review of included studies found large heterogeneity of participants, diverse settings, and different methods of measuring variables. A descriptive synthesis was deemed more appropriate and undertaken using the FORM framework to grade the strength of recommendations based on evidence base, consistency, clinical impact and generalisability [25]. Studies’ level of evidence was based on the Australian National Health and Medical Research Council (NHMRC) hierarchy of evidence designated by the type of research question [26].

## Results

### Search results

**Fig 1:**
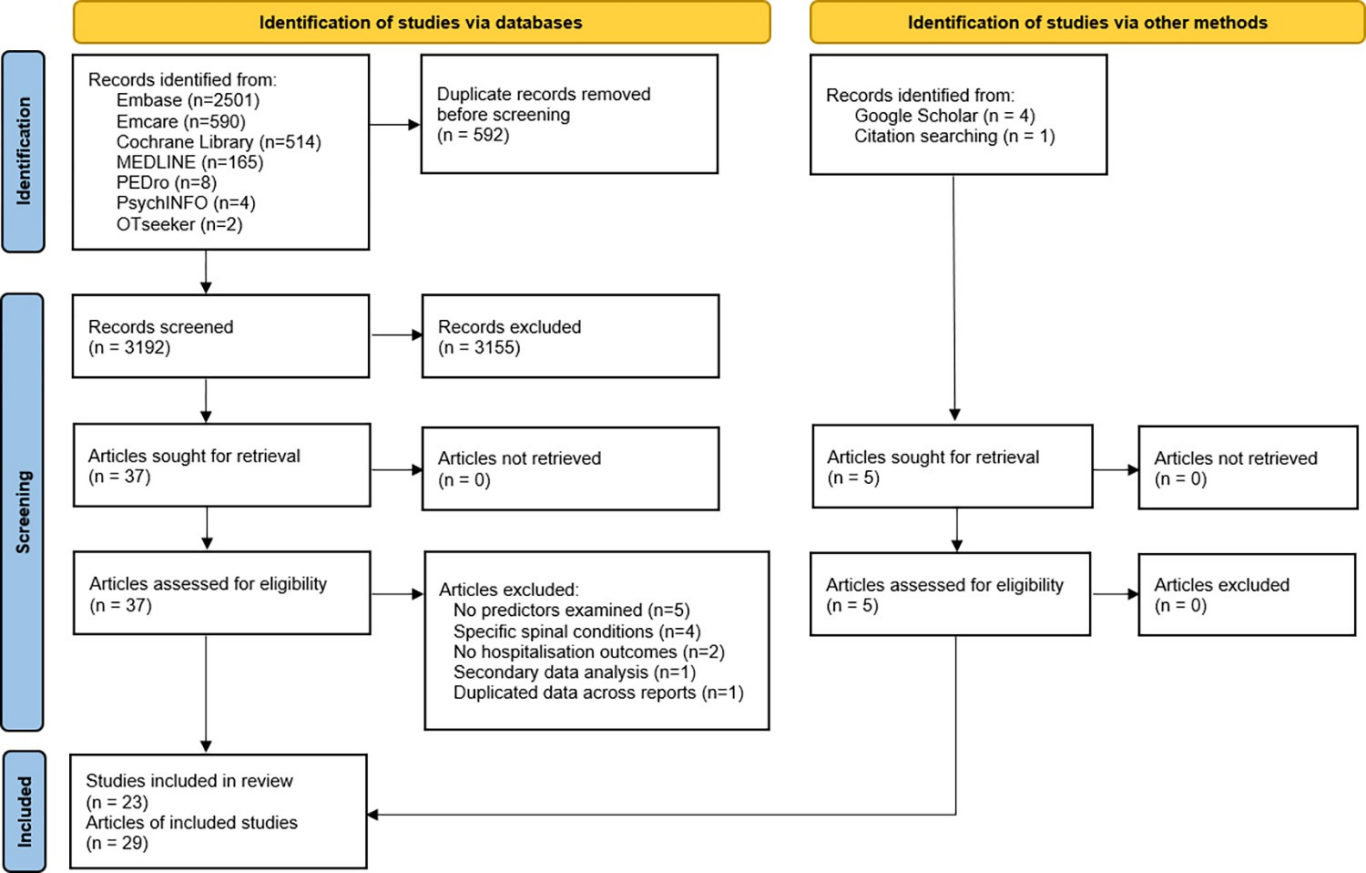
PRISMA flow diagram [21].

The initial search identified 3784 records. After pooling searches and removing duplicates, titles and abstracts were screened leaving 37 potential articles. Grey literature search and searching through reference lists included five additional articles. The full texts were retrieved and assessed for eligibility and 23 studies published over 29 articles were identified as being eligible for review. The literature selection process is outlined in Fig 1.

### Study characteristics

Table 1 provides an overview of the included articles. They were published between 1987 and 2022. Studies that were published over multiple articles examined unique predictors of hospitalisation without duplication of outcomes and are therefore presented separately in this review. From the 23 studies, there were five prospective cohort studies, fifteen retrospective cohort studies, two retrospective case-control studies, and one cross-sectional study. Two types of research questions were reported: prognostic and aetiology. Studies with prognostic research question types examined the factors that predict hospitalisation in populations with LBP at baseline, and included three Level II, twelve Level III-3, and one Level IV studies on the NHMRC hierarchy of evidence. Aetiology research questions examined the causalty of hospitalisation in populations that did not have LBP at baseline, and included three Level II, two Level III-2 and two Level III-3 studies on the NHMRC hierarchy of evidence.

**Table 1:** Summary of included articles.

Article	Design & level	Participants	Methodology	Predictors investigated		Outcomes	Significance
	of evidence^∆^			Variables	Comparators		
Anderson et al. 2022 Australia [27]	Retrospective cohort Level III-3 prognostic	1,339,209 adults with LBP 52% female 50.7 years average age 30% hospitalised	Audit of hospital databases SNOMED-CT and ICD 9 codes for LBP Data capture period 10 years Follow-up not applicable	Male Age 25–44 years Age 45–64 years Age 65–84 years Age +85 years Attended ED via ambulance Privately referred High socioeconomic Compensable: work injury Compensable: motor vehicle Compensable: overseas Compensable: defence force	Female 0–24 years 0–24 years 0–24 years 0–24 years Walked in Self-referred Low socioeconomic Not compensable Not compensable Not compensable Not compensable	OR 1.0, 95% CI 0.99–1.01 OR 1.30, 95% CI 1.28–1.32 OR 1.90, 95% CI 1.87–1.94 OR 3.81, 95% CI 3.74–3.88 OR 5.45, 95% CI 5.34–5.57 OR 4.12, 95% CI 3.98–4.26 OR 2.18, 95% CI 2.06–2.30 OR 1.26, 95% CI 1.24–129 OR 0.42, 95% CI 0.40–0.43 OR 0.90, 95% CI 0.86–0.94 OR 0.74, 95% CI 0.68–0.81 OR 1.29, 95% CI 1.04–1.59	* * * * * * * * * * *
Beyera et al. 2020 Ethiopia [28]	Cross-sectional study Level IV prognostic	543 adults with LBP 38% female 43 years average age 14% hospitalised 7 days average stay	Interviews Systematic random sampling Self-reported LBP Data capture period 6 months Follow-up not applicable	Female Age 30–39 years Age 40–49 years Age +50 years Non-alcohol consumer Non-smoker Education university level Reside in rural area Residence alone Radiating leg pain Pain intensity moderate Pain intensity severe Self-reported health poor Insomnia Depressive symptoms Negative beliefs about LBP	Male 18–29 years 18–29 years 18–29 years Alcohol consumer Smoker No formal education Reside in urban area Reside with family No leg pain Pain intensity mild Pain intensity mild Self-reported excellent No insomnia No depressive symptoms Positive beliefs about LBP	PR 1.8, 95% CI 1.2–2.75 PR 3.0, 95% CI 1.2–10.0 PR 3.5, 95% CI 1.4–11.7 PR 4.3, 95% CI 1.8–14.1 PR 0.58, 95% CI 0.37–0.91 PR 0.43, 95% CI 0.21–1.42 PR 1.45, 95% CI 0.79–2.81 PR 0.55, 95% CI 0.34–0.9 PR 2.54, 95% CI 1.34–4.15 PR 1.34, 95% CI 0.89–1.95 PR 2.46, 95% CI 1.15–5.52 PR 8.84, 95% CI 4.82–18.13 PR 1.72, 95% CI 0.71–6.48 PR 1.40, 95% CI 0.96–2.01 PR 1.09, 95% CI 0.68–1.68 PR 1.16, 95% CI 0.79–1.75	* * * * * * * * *
Buchbinder et al. 2022 Australia [29]	Retrospective cohort Level III-3 prognostic	450 adults with LBP Attending an ED 60% female 69 years age average 53% hospitalised	Audit of hospital records ICD 10 codes for LBP Data capture period 1 year Follow-up not applicable	Age: for every 1 year of age Attended ED via ambulance Time spent in ED Pathology tests in ED CT lumbar spine in ED	Continuous data^#^ Attended via private car Continuous data^#^ No pathology tests No CT lumbar spine	OR 1.03. 95% CI 1.02–1.05 OR 2.03, 95% CI 1.06–3.90 OR 1.16, 95% CI 1.07–1.26 OR 3.32, 95% CI 2.01–5.49 OR 1.86, 95% CI 1.12–3.11	* * * * *
Davidson et al. 2022 Australia [30]	Retrospective cohort Level III-3 prognostic	26,509 adults with LBP Attending an emergency department 52% female 49.2 years age average, SD 20.0 18% hospitalised	Audit of hospital databases ICD 10 codes for LBP Data capture period 5 years Follow-up not applicable	Major city hospital Outer regional hospital ED specialty classification ED specialist physician	Inner regional hospital Inner regional hospital Lower classification Visiting medical officer	OR 1.36, 95% CI 1.27–1.46 OR 1.23, 95% CI 1.12–1.35 OR 1.22, 95% CI 1.14–1.30 OR 1.0, 95% CI 0.93–1.07	* * *
de Heer et al. 2016 USA [31]	Retrospective cohort Level III-3 prognostic	413,608 adults with LBP attending community clinics or hospital outpatients. 65% female 70 years age average, SD 13.4 3.7% hospitalised	Random sample of Medicare claims ICD 9 codes for LBP Data capture period 1 year Follow-up period 180-days	Physical therapy: At 30 days At 180 days	No physical therapy	RR 0.35, 95% CI 0.30–0.40 RR 0.68, 95% CI 0.64–0.72	* *
Euro et al. 2018 Finland [32]	Retrospective cohort Level III-2 aetiology	13,094 general adult population attending a community clinic. 41% female 20–59 years age range 5.3% hospitalised	Survey and examination Audit of hospital databases ICD 8–10 codes for sciatica Data capture period 4 years Follow-up period 34 years	*Women*: Age 30–39 years Age 40–49 years Age 50–59 years Body height >170 cm BMI >30 kg/m^2^ Self-reported health poor Smoker Leisure time physically active Nurse Sales worker Industry worker *Men*: Age 30–39 years Age 40–49 years Age 50–59 years Body height >180 cm BMI >30 kg/m^2^ Self-reported health poor Smoker Leisure time physically active Metal/machine worker Other industrial worker	20–29 years 20–29 years 20–29 years <160 cm <25 kg/m^2^ Good Non-smoker Inactive White collar worker White collar worker White collar worker 20–29 years 20–29 years 20–29 years <170 cm BMI <25 kg/m^2^ Self-reported good Non-smoker Inactive White collar worker White collar worker	HR 1.27, 95% CI 0.96–1.66 HR 0.85, 95% CI 0.61–1.18 HR 0.60, 95% CI 0.40–0.91 HR 1.69, 95% CI 1.12–2.54 HR 1.27, 95% CI 0.86–1.88 HR 1.75, 95% CI 1.02–2.98 HR 1.78, 95% CI 1.19–2.66 HR 0.90, 95% CI 0.62–1.30 HR 1.81, 95% CI 1.18–2.78 HR 1.56, 95% CI 1.05–2.31 HR 1.46, 95% CI 1.03–2.08 HR 1.05, 95% CI 0.80–1.37 HR 0.95, 95% CI 0.70–1.28 HR 0.59, 95% CI 0.38–0.90 HR 1.16, 95% CI 0.83–1.62 HR 1.03, 95% CI 0.68–1.56 HR 2.00, 95% CI 1.32–3.03 HR 1.17, 95% CI 0.88–1.56 HR 0.74, 95% CI 0.55–1.00 HR 2.57, 95% CI 1.47–4.50 HR 1.44, 95% CI 1.06–1.95	* * * * * * * * * * *
Euro et al. 2019 Finland [33]	Retrospective cohort Level III-2 aetiology	3891 general adult population attending a community clinic. 51% female 30–59 years age range 3.1% hospitalised	Survey and examination Audit of hospital databases ICD 8–10 codes for sciatica Data capture period 3 years Follow-up period 30 years	Age 40–49 years Age 50–59 years Female BMI >30 kg/m^2^ Self-reported health poor Smoker Leisure time physically active Education level <8 years Occupation sedentary Occupation heavy work Occupation lifting tasks Occupation awkward postures Occupation prolonged sitting Occupation vibration	30–39 years 30–39 years Male BMI <25 kg/m^2^ Self-reported good Non-smoker Inactive Education >12 years Heavy work Sedentary work No lifting No awkward postures No prolonged sitting No vibration exposure	HR 0.65, 95% CI 0.43–0.96 HR 0.23, 95% CI 0.12–0.46 HR 0.87, 95% CI 0.57–1.35 HR 1.39, 95% CI 0.78–2.48 HR 1.25, 95% CI 0.50–3.11 HR 1.49, 95% CI 0.97–2.28 HR 0.90, 95% CI 0.54–1.48 HR 0.72, 95% CI 0.39–1.34 HR 1.57, 95% CI 1.05–2.34 HR 0.48, 95% CI 0.26–0.89 HR 2.1, 95% CI 1.35–3.26 HR 0.68, 95% CI 0.43–1.03 HR 1.14, 95% CI 0.76–1.71 HR 1.61, 95% CI 0.95–2.72	* * * * *
Ferreira et al. 2019 Australia [34]	Retrospective cohort Level III-3 prognostic	6393 adults with LBP attending ED. 50% female 52.4 years age average (SD 20.3) 17.6% hospitalised 6 days average stay	Audit of hospital records SNOMED-CT codes for LBP Data capture period 2.5 years Follow-up not applicable	Age ≥65 years Non-serious spinal pathology Attended ED via ambulance Attended ED day hours ED triage score urgent ED triage score semi-urgent	Age <65 years Serious spinal pathology Attended in private car Attended out of hours Non-urgent Non-urgent	OR 3.0, 95% CI 2.59–3.59 OR 0.23, 95% CI 0.17–0.32 OR 2.98, 95% CI 2.53–3.51 OR 1.74, 95% CI 1.48–2.05 OR 3.37, 95% CI 1.48–9.38 OR 2.99, 95% CI 1.37–6.48	* * * * * *
Ferreira et al. 2022 Australia [35]	Retrospective cohort Level III-3 prognostic	176.726 adults with LBP attending an ED (n = 177) 52% female 51.8 years age average (SD 19.5) 25.2% hospitalised	Audit of hospital databases SNOMED-CT and ICD-9, 10 codes for LBP Data capture period 4 years Follow-up not applicable	Hospital contextual factors	No specific comparator, but observed variations in hospitalisations across hospitals attributed to contextual factors controlling for patient and case-mix factors	Hospital factors explained 10% of variation in hospitalisations (ICC = 0.10). The median OR was 2.03 if attending a different hospital with higher hospitalisation rates.	*
Heliövaara et al. 1987 Finland [36]	Retrospective case-control Level III-3 aetiology	57,000 general adult population attending a community clinic. Unknown % female 20–59 years age range 2.7% hospitalised	Survey Audit of hospital databases ICD 8 codes for LBP or sciatica Data capture period 7 years Follow-up period 11 years	Stress symptoms Frequent analgesia use Smoker Leisure time physically active Middle social class Marital status single Industry worker Sales worker Agriculture/forestry worker	No stress symptoms No analgesia use Non-smoker Inactive Upper class Married White collar worker White collar worker White collar worker	RR = 2.1, CI nil, p<0.001 RR = 2.1, 95% CI 1.4–3.2 No predictive value No predictive value RR = 4.0, 95% CI 2.3–6.8 No predictive value RR = 1.9, 95% CI 1.3–2.7 RR = 2.2, 95% CI 1.4–3.4 RR = 1.5, 95% CI 0.9–2.5	* * * * *
Joines et al. 2003 USA [37]	Retrospective cohort Level III-3 prognostic	15,107 adults with LBP admitted as a hospital inpatient Unknown % female Unknown age details 100% hospitalised	Audit of hospital databases ICD 9 codes for LBP Data capture period 3 years Follow-up not applicable	Non-white ethnicity Disability Unemployed Education level graduate Reside in urban area Median household income Occupation lifting/transport Hospital bed occupancy Primary care physician density Spinal surgeon density Chiropractor density Discharge rate, other causes Hospital MRI/CT availability	Predictors treated as continuous variables in the regression analysis, therefore no comparison is made ^#^	A spatial lag model (R^2^) that provides a weighted average of observed variables at neighbouring locations, explained 70% of hospitalisations for LBP (p<0.001).	* * * * * *
Jørgensen et al. 2013^ǂ^ Denmark [38]	Prospective cohort Level II aetiology	3833 general adult population attending work health assessment 0% female 48 years age average 1.67% hospitalised	Survey and examination Audit of hospital databases ICD 8–10 codes for lumbar disc disorders Data capture period 2 year Follow-up period 31 years	Fitness VO_2_ max high	VO_2_ max low	HR 0.88, 95% CI 0.51–1.50	
Kääriä et al. 2005 ^ʃ^ Finland [39]	Prospective cohort Level II aetiology	902 general adult population attending work health assessment 32% female 17–65 years age range 5.7% hospitalised	Survey and examination Audit of hospital databases ICD 8 codes for LBP 27 years observation time	Male Blue collar worker Work absenteeism Radiating leg pain Pain distress symptoms Examination findings Past history of LBP	Female White collar worker No work absenteeism No leg pain No pain distress No findings No history of LBP	HR 0.46, 95% CI 0.26–0.79 HR 2.29, 95% CI 1.23–4.24 HR 3.3, 95% CI 1.6–6.7 HR 3.7, 95% CI 1.8–7.7 HR 3.69, 95% CI 1.51–9.05 HR 2.4, 95% CI 1.3–4.7 HR 2.8, 95% CI 1.5–5.4	* * * * * * *
Kääriä et al. 2014 ^ʃ^ Finland [40]	Prospective cohort Level II aetiology	902 general adult population attending work health assessment 32% female 17–65 years age range 8.3% hospitalised	Survey and examination Audit of hospital databases ICD 8–10 codes for disc disorders Data capture period 1 year Follow-up period 27 years	High leisure physical activity Some leisure physical active	Low activity Low activity	HR 0.40, 95% CI 0.21–0.79 HR 0.76, 95% CI 0.44–1.29	*
Kaila-Kangas et al. 2003 ^ʃ^ Finland [41]	Prospective cohort Level II aetiology	902 general adult population attending work health assessment 32% female 17–65 years age range 8.3% hospitalised	Survey and examination Audit of hospital databases ICD 8–10 codes for disc disorders Data capture period 1 year Follow-up period 27 years	BMI >27.5 kg/m^2^ Smoker	BMI <25 kg/m^2^ Non-smoker	RR 2.7, 95% CI 1.1–6.5 RR 3.4, 95% CI 1.3–9.0	* *
Kaila-Kangas et al. 2004 ^ʃ^ Finland [42]	Prospective cohort Level II aetiology	902 general adult population attending work health assessment 32% female 17–65 years age range 8.3% hospitalised	Survey and examination Audit of hospital databases ICD 8–10 codes for disc disorders Data capture period 1 year Follow-up period 27 years	Low job control/flexibility Low supervisor support Low co-worker support High job demands General stress symptoms	High job control/flexibility High supervisor support High co-worker support Low job demands No stress symptoms	RR 3.2, 95% CI 1.3–7.8 RR 2.9, 95% CI 1.3–6.3 RR 0.76, 95% CI 0.36–6.32 RR 0.9, 95% CI 0.4–1.9 RR 1.3, 95% CI 0.5–3.0	* *
Kaila-Kangas et al. 2006 ^ʃ^ Finland [43]	Prospective cohort Level II aetiology	902 general adult population attending work health assessment 32% female 17–65 years age range 8.3% hospitalised	Survey and examination Audit of hospital databases ICD 8–10 codes for disc disorders Data capture period 1 year Follow-up period 27 years	Self-reported sleep disturbance	No sleep disturbance	RR 2.4, 95% CI 1.2–4.6	*
Kawchuk et al. 2022 Canada [44]	Prospective cohort Level II prognostic	209 adults with LBP Attending an emergency department 50% female 49 years age average, IQR 35–66 9% hospitalised	Questionnaire Audit of hospital databases Self-reported LBP Data capture period 1 year Follow-up not applicable	Male Age: for every 1 year of age Attended ED via ambulance Negative beliefs about LBP	Female Continuous data^#^ Private vehicle/ walked in Continuous data^#^	OR 1.51, 95% CI 0.54–4.21 OR 1.05, 95% CI 1.02–1.08 OR 4.95, 95% CI 1.79–13.7 Urgency of pain, impact on function, perceived quality of care from ED, expectation for admission were higher in those hospitalised with LBP.	* * *
Leino-Arjas et al. 2002 Finland [45]	Retrospective cohort Level III-3 prognostic	1,742 adults with LBP admitted as a hospital inpatient 48% female 20–64 years age range 100% hospitalised	Audit of hospital databases ICD 10 codes for lumbar disc disorders Data capture period 1 year Follow-up not applicable	*Women*: Education level low Low income Labourer, specialised Labourer, non-specialised *Male*: Education level low Low income Labourer, specialised Labourer, non-specialised	Higher education High income White collar worker White collar worker Higher education High income White collar worker White collar worker	RR 2.47, 95% CI 1.99–3.06 RR 1.34, 95% CI 1.01–1.79 RR 2.66, 95% CI 2.00–3.55 RR 3.33, 95% CI 2.41–4.60 RR 1.86, 95% CI 1.61–2.16 RR 0.87, 95% CI 0.65–1.17 RR 2.51, 95% CI 1.95–3.23 RR 2.82, 95% CI 2.14–3.72	* * * * * * *
Mattila et al. 2008 Finland [46]	Prospective cohort Level II prognostic	57,408 adolescents, population-based cohort 54% female 14–18 years age range 1.1% hospitalised	Audit of hospital databases ICD 8–10 codes for LBP or sciatica Data capture period 18 years Follow-up period 11 years	Male Childhood health complaints Childhood smoker Childhood rural residence School success poor	Female No health complaints Childhood non-smoker Childhood city residence School success good	HR 3.2, 95% CI 2.7–3.7 HR 1.5, 95% CI 1.2–1.9 HR 1.4, 95% CI 1.1–1.7 HR 1.3, 95% CI 0.9–1.8 HR 1.4, 95% CI 1.1–1.9	* * * *
Ritzwoller et al. 2006 USA [47]	Retrospective cohort Level III-3 prognostic	16,567 adults with LBP attending hospital outpatients or inpatients 54% female 51 years average age 2.6% hospitalised	Audit of hospital databases ICD 9 codes for LBP Data capture period 2 years Follow-up period 2 years	Male Age 65–74 years Age 75–85 years Age >85 years Opioid use prior to LBP NSAIDs use prior to LBP Anxiety Psychosis Depression Asthma/ COPD Diabetes Gastrointestinal disorders Heart disease/ HTN Rheumatoid arthritis Past history of LBP	Female Age 18–24 years Age 18–24 years Age 18–24 years No opioid use No NSAIDs use No anxiety No psychosis No depression No asthma/COPD No diabetes No gastrointestinal disorder No heart disease/ HTN Np rheumatoid arthritis No history of LBP	OR 1.22, 95% CI 0.99–1.49 OR 5.11, 95% CI 2.04–12.76 OR 11.26, 95% CI 4.50–28.21 OR 12.57, 95% CI 4.67–34.16 OR 1.27, 95% CI 1.02–1.59 OR 1.76, 95% CI 1.43–2.17 OR 1.17, 95% CI 0.89–1.53 OR 1.38, 95% CI 0.81–2.35 OR 0.96, 95% CI 0.74–1.27 OR 1.22, 95% CI 0.92–1.60 OR 1.14, 95% CI 0.80–1.63 OR 1.10, 95% CI 0.86–1.43 OR 1.19, 95% CI 0.95–1.49 OR 1.11, 95% CI 0.74–1.65 6 times more likely, p<0.001	* * * * * *
Rivinoja et al. 2011 Finland [48]	Prospective cohort Level II prognostic	9,016 adolescents 50% female 14 years ago average 2.1% hospitalised	Survey Audit of hospital databases ICD 8–10 codes for LBP or sciatica Data capture period 1 year Follow-up period 15 years	*Girls*: Overweight/obese childhood Played childhood sport Childhood smoker *Boys*: Overweight/obese childhood Played childhood sport Childhood smoker	Normal weight childhood Minimal childhood sport Childhood non-smoker Normal weight childhood Minimal childhood sport Childhood non-smoker	HR 1.9, 95% CI 0.9–3.9 HR 1.4, 95% CI 0.8–2.3 HR 1.5, 95% CI 0.9–2.6 HR 0.9, 95% CI 0.5–1.7 HR 0.9, 95% CI 0.6–1.3 HR 1.18 95% CI 1.2–2.7	*
Sayer et al. 2018 Australia [49]	Retrospective cohort Level III-3 prognostic	1089 adults with LBP attending ED 50% female 42 years average range, 30–54 range 27.0% hospitalised	Audit of hospital records ICD 10 codes for LBP or sciatica Data capture period 1 year Follow-up not applicable	Female ED clinician doctor/nurse	Male ED clinician physiotherapist	OR 1.46, 95% CI 1.15–1.85 OR 3.38, 95% CI 2.33–4.91	* *
Sørensen et al. 2011 ^ǂ^ Denmark [50]	Prospective cohort Level II aetiology	3833 general adult population attending work health assessment 0% female 48 years age average 1.7% hospitalised	Survey and examination Audit of hospital databases ICD 8–10 codes for lumbar disc disorders Data capture period 2 years Follow-up period 31 years	Body height 172–177 cm Body height >178 cm Body weight 73–80 kg Body weight >81 kg Sedative use prior to LBP Smoker Alcohol 3–5 drinks/day Medium physical activity High physical activity Middle social class Lower social class Mental stress at work General stress symptoms Occupation light demands Occupation strenuous	Body height <171 cm Body height <171 cm Body weight <72 kg Body weight <72 kg No sedative use Non-smoker Non-alcohol consumer Low physical activity Low physical activity Upper social class Upper social class No work mental stress No general stress Seldom physical Seldom physical	HR 2.18, 95% CI 1.10–4.32 HR 1.88, 95% CI 0.92–3.86 HR 1.40, 95% CI 0.69–2.86 HR 1.81, 95% CI 0.89–3.66 HR 1.08, 95% CI 0.58–2.00 HR 0.95, 95% CI 0.40–2.23 HR 1.03, 95% CI 0.47–2.26 HR 0.72, 95% CI 0.38–1.97 HR 0.78, 95% CI 0.31–1.97 HR 1.58, 95% CI 0.65–3.82 HR 2.22, 95% CI 0.87–5.65 HR 0.64, 95% CI 0.32–1.26 HR 0.47, 95% CI 0.11–1.94 HR 2.37, 95% CI 1.36–4.13 HR 3.91, 95% CI 1.82–8.38	* * *
Urrutia et al. 2020 Chile [51]	Retrospective cohort Level III-3 prognostic	519 adults with LBP attending ED 58% female 44 years average age, SD 16.6 3.1% hospitalised	Audit of medical records LBP diagnosis on discharge Data capture period 6 months Follow-up not applicable	Age Pain intensity: each point Pain duration Previous ED LBP encounter Neurological impairments Red flag present	Continuous data^#^ Continuous data^#^ Continuous data^#^ No past ED LBP encounter No neuro impairment Red flags absent	OR 0.98, 95% CI 0.93–1.70 OR 1.95, 95% CI 1.14–3.33 OR 1.01, 95% CI 0.99–1.05 OR 6.57, 95% CI 0.84–51.51 OR 4.41, 95% CI 0.2–96.61 OR 2.76, 95% CI 0.21–35.91	*
Videman et al. 1995 Finland [52]	Retrospective case-control Level III-3 prognostic	937 former elite athletes 620 healthy controls 0% female 45–64 years age range 1.4–8% hospitalised	Survey Audit of public and hospital records ICD 8–9 codes for LBP or sciatica Data capture period 45 years Follow-up period 17 years	Age: for every 1 year of age BMI >30 kg/m^2^ Body height Life dissatisfaction Personality: extroversion Personality: neuroticism Personality: hostility Sleep disturbance Smoker Alcohol consumption, much High leisure physical activity Education level low Divorced/widowed Unemployed/pension Occupation heavy loads Monotonous work	Continuous data^#^ BMI ≤30 kg/m^2^ Continuous data^#^ Life satisfaction Personality trait absent Personality trait absent Personality trait absent No sleep disturbance Non-smoker Non-alcohol consumption Low leisure physical activity Education level high Married Employed Not heavy load Not monotonous work	OR 1.0 OR 1.0 OR 1.0 OR 1.3 OR 0.8 OR 0.5 OR 1.6 OR 0.5 OR 1.3 OR 0.8 OR 1.4 OR 1.5 OR 0.8 OR 3.0, 95% CI 2.59–3.59 OR 2.0 OR 1.1	*
Wahlström et al. 2012 ^˄^ Sweden [53]	Prospective cohort Level II aetiology	263,529 general adult population attending work health assessment 0% female 20–65 years age range 1% hospitalised	Survey and examination Audit of hospital databases ICD 9–10 codes for lumbar disc disorders Data capture period 22 years Follow-up period 17 years	Body height >190 cm Body weight >100 kg Age 30–39 years Age 40–49 years Age 50–59 years Age 60–65 years Manual labourer Smoker	170–179 cm 70–89 kg 20–29 years 20–29 years 20–29 years 20–29 years White collar worker Non-smoker	RR 1.55. 95% CI 1.30–1.86 RR 1.40, 95% CI 1.12–1.76 RR 1.87, 95% CI 1.30–1.86 RR 1.75, 95% CI 1.47–2.08 RR 1.08, 95% CI 0.90–1.31 RR 0.86, 95% CI 0.68–1.09 RR 1.55, 95% CI 1.29–1.87 RR 1.27, 95% CI 1.15–1.39	* * * * *
Wahlström et al. 2018 ^˄^ Sweden [54]	Prospective cohort Level II aetiology	288,926 general adult population attending work health assessment 0% female 30–49 years age average 1% hospitalised	Survey and examination Audit of hospital databases ICD 9–10 codes for lumbar disc disorders Data capture period 22 years Follow-up period 24 years	Occupation moderate to high vibration exposure	No or low vibration exposure	RR 1.35, 95% CI 1.12–1.63	*
Whedon et al. 2022 USA [55]	Retrospective cohort Level III-3 prognostic	28,160 older adults with chronic LBP Attending outpatient clinics 70% female 65–84 years age range 4% hospitalised	Sample of Medicare claims ICD 9–10 codes for LBP Data capture period 1 year Follow-up period 4 years	Initiated long term-opioid therapy	Initiated long-term spinal manipulative therapy (with cross overs)	OR 3.64, 95% CI 2.92–4.53	*

^ǂ ʃ ˄^ Refers to studies with similar baseline data published over multiple articles.

^∆^ National Health and Medical Research Council hierarchy of evidence designated by the research question [26].

^#^ Predictors are treated as continuous data in the analysis without a comparator variable.

* Significant association: statistical significance p<0.05 and/or confidence interval not spanning 1.

CI confidence interval, HR hazard ratio, OR odd ratio, PR prevalence ratio, RR relative risk reduction, SD standard deviation.

BMI body mass index, COPD chronic obstructive pulmonary disease, ED emergency department, HTN hypertension, ICD International Classification of Diseases, LBP low back pain, CT computed topography, MRI magnetic resonance imaging, NSAIDs nonsteroidal anti-inflammatory drugs, SNOMED-CT Systemized Nomenclature of Medicine Clinical Terms, VO_2_ max maximal oxygen consumption.

The majority of studies were conducted in Finland (n = 8), followed by Australia (n = 6), the United States of America (n = 4), Canada (n = 1), Chile (n = 1), Denmark (n = 1), Ethiopia (n = 1) and Sweden (n = 1). Studies were set across a range of settings including emergency departments (n = 8), community and outpatient clinics (n = 5), hospital inpatients (n = 2), hospital inpatients and outpatients (n = 1), workplaces (n = 3) and the remainder were population-based studies (n = 4). The total number of participants across studies included 2,026,834 with LBP and 435,663 without LBP pain at baseline. Most studies excluded or unequally represented women, especially in studies set in workplaces where the majority of the workforce comprised men. Two studies included adolescents at baseline but the hospitalisations occurred almost exclusively in adulthood [46, 48].

All studies undertook audits of hospital records and databases except the cross-sectional study that used interviews and self-reported outcomes [28]. The International Classification of Diseases (ICD) or the Systematized Nomenclature of Medicine—Clinical Terms (SNOMED-CT) primary discharge diagnoses for non-specific LBP conditions or sciatica were used for the inclusion criteria in all but three studies, where participants self-reported their LBP [28, 44, 51]. Data capture period or participant recruitment ranged from six months to 45 years, and follow-up of participants ranged between 180 days and 34 years from baseline. Rates of hospitalisation for LBP ranged from 1–2% in population-based studies, 1–5% in community and outpatient clinics, 1–8% in workplaces, and 3–53% in emergency departments. Details of hospital length of stay were available in three studies with averages ranging from two to seven days [28, 30, 34].

**Fig 2:**
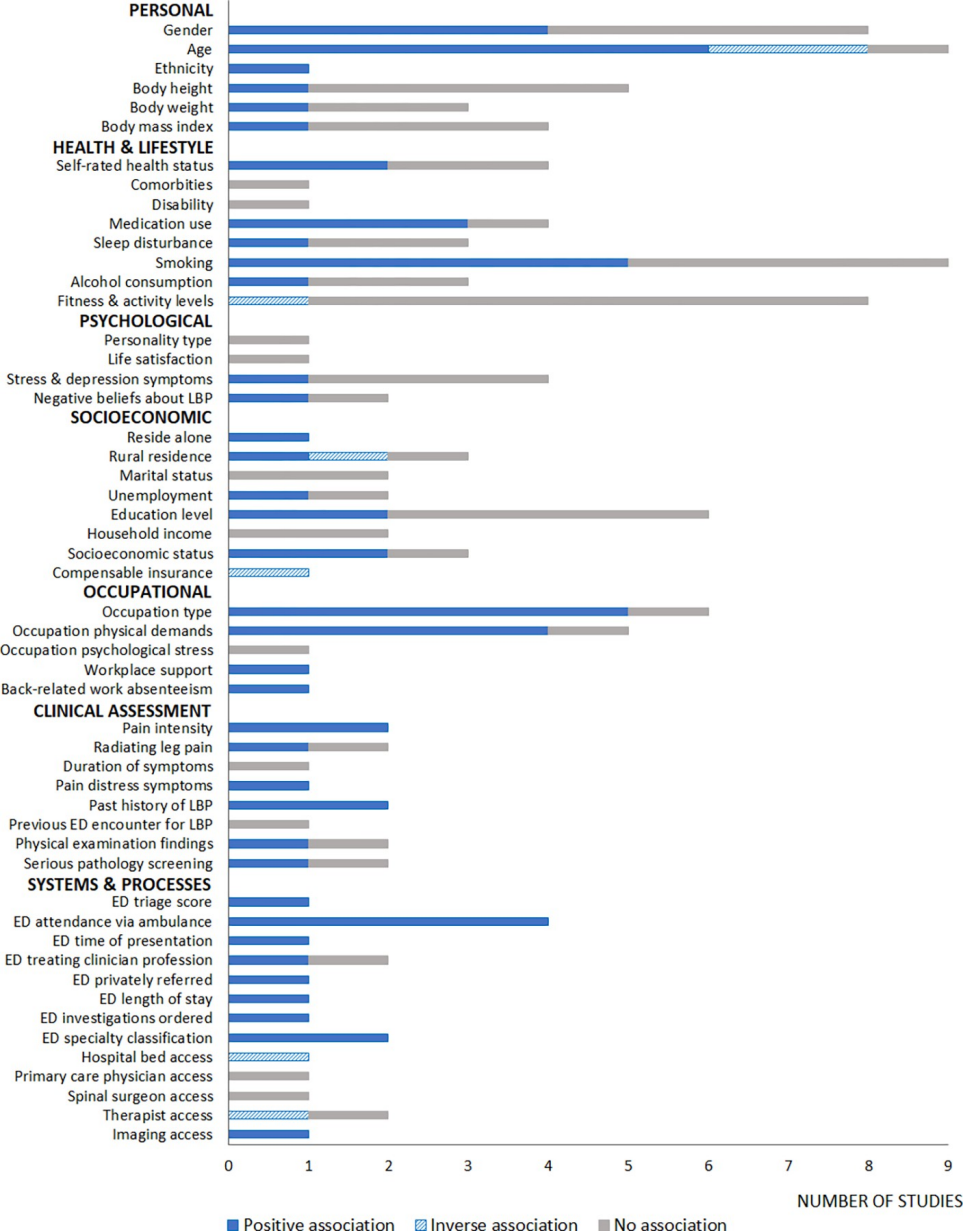
Number of articles investigating each predictor variable and the associations with hospitalisation for LBP.

There were 52 predictor variables investigated across all studies. They have been categorised into personal factors, health and lifestyle factors, psychological factors, socioeconomic factors, occupational factors, clinical assessment findings, and health systems and processes. These categories have been based on previous reviews supporting a biopsychosocial framework for LBP [56–58]. Fig 2 illustrates the number of articles found examining each variable and its association with hospitalisation for LBP. The following sections summarise the descriptive analysis for each category.

### Risk of bias assessment

The results of the risk of bias assessment are outlined in Tables 2 and 3. Of the 29 articles, six were considered low risk, thirteen of moderate risk and ten of high risk of bias. Large sample sizes, objective measures of hospitalisation outcomes, and identification and inclusion of important confounding factors into analyses were strengths across most articles. Long follow-up periods were also considered a strength, however certain predictor variables at baseline, such as smoking status or body weight are not fixed conditions and therefore accuracy in measuring such variables over time is uncertain impacting the believability of results. Various predictor variables relied on invalid, subjective measures also impacting the believability of results. Generalisability of results was often limited by unequal representation of genders amongst participants and a focus on specific populations, such as occupation types. There were some inconsistencies between study findings and previously published literature likely reflecting overall lack of methodological rigor in studies. Practice implications were also poorly addressed.

**Table 2:** Risk of bias assessment of included studies based on the Critical Appraisal Skills Programme Cohort Study Checklist [23].

Article	1	2	3	4	5	6	7	8	9	10	11	12	Risk Assessment
Anderson et al. 2022 [27]	Y	Y	Y	Y	Y	n/a	n/a	Y	Y	Y	Y	Y	Low
Beyera et al. 2020 [28]	Y	Y	Y	?	Y	n/a	n/a	Y	Y	?	Y	Y	Moderate
Buchbinder et al. 2022 [29]	Y	Y	Y	Y	?	n/a	n/a	Y	Y	?	Y	Y	Moderate
Davidson et al. 2022 [30]	Y	Y	Y	Y	?	n/a	n/a	?	Y	Y	Y	Y	Moderate
de Heer et al. 2016 [31]	Y	Y	Y	Y	Y	N	n/a	Y	?	?	Y	N	High
Euro et al. 2018 [32]	Y	Y	Y	Y	Y	Y	n/a	Y	?	Y	Y	N	Moderate
Euro et al. 2019 [33]	Y	Y	Y	Y	Y	Y	n/a	Y	?	Y	Y	N	Moderate
Ferreira et al. 2019 [34]	Y	Y	Y	Y	Y	n/a	n/a	Y	Y	Y	Y	Y	Low
Ferreira et al. 2022 [35]	Y	Y	Y	Y	Y	n/a	n/a	Y	Y	Y	?	Y	Low
Joines et al. 2003 [37]	Y	Y	Y	Y	Y	Y	n/a	Y	Y	Y	Y	Y	Low
Jørgensen et al. 2013 [38]	Y	Y	Y	Y	Y	Y	n/a	N	Y	?	?	Y	Moderate
Kääriä et al. 2005 [39]	Y	?	Y	Y	Y	Y	n/a	Y	?	?	Y	Y	Moderate
Kääriä et al. 2014 [40]	Y	?	?	Y	Y	Y	n/a	Y	?	?	?	?	High
Kaila-Kangas et al. 2003 [41]	Y	Y	Y	Y	Y	Y	n/a	Y	?	?	Y	?	Moderate
Kaila-Kangas et al. 2004 [42]	Y	Y	Y	Y	Y	Y	n/a	Y	?	?	Y	?	Moderate
Kaila-Kangas et al. 2006 [43]	Y	Y	?	Y	Y	Y	n/a	Y	?	?	?	Y	High
Kawchuk et al. 2022 [44]	Y	?	?	Y	Y	n/a	n/a	Y	?	Y	Y	Y	Moderate
Leino-Arjas et al. 2002 [45]	Y	?	?	Y	Y	Y	n/a	Y	?	?	?	?	High
Mattila et al. 2008 [46]	Y	Y	?	Y	Y	Y	n/a	Y	?	?	Y	?	High
Ritzwoller et al. 2006 [47]	Y	Y	Y	?	Y	Y	n/a	?	Y	Y	?	Y	Moderate
Rivinoja et al. 2011 [48]	Y	Y	?	Y	Y	Y	n/a	N	N	N	?	Y	High
Sayer et al. 2018 [49]	Y	Y	Y	Y	Y	n/a	n/a	Y	?	Y	Y	Y	Low
Sørensen et al. 2011 [50]	Y	Y	?	Y	Y	Y	n/a	Y	?	?	?	N	High
Urrutia et al. 2020 [51]	Y	Y	?	?	Y	n/a	n/a	Y	?	Y	Y	Y	Moderate
Wahlström et al. 2012 [53]	Y	Y	?	Y	Y	Y	n/a	Y	Y	?	Y	N	Moderate
Wahlström et al. 2018 [54]	Y	Y	Y	Y	Y	Y	n/a	Y	Y	?	Y	Y	Low
Whedon et al. 2022 [55]	Y	?	?	Y	Y	Y	n/a	Y	Y	?	Y	?	High

Y = yes (green shading);? = unclear (amber shading); N = no (red shading); n/a = not applicable (grey shading).

1. Focused issue; 2. Cohort recruitment; 3. Exposure accurately measured; 4. Outcome accurately measured; 5. Confounders identified and included in analysis; 6. Follow-up complete and adequate length; 7. Results reported; 8. Statistical precision; 9. Believability of results; 10. Generalisability; 11. Results consistent with other evidence; 12. Practice implications.

**Table 3:** Risk of bias assessment of included studies based on the Critical Appraisal Skills Programme Case Control Study Checklist [24].

Article	1	2	3	4	5	6	7	8	9	10	11	Risk Assessment
Heliövaara et al. 1987 [36]	Y	Y	Y	Y	N	Y	N	N	?	?	?	High
Videman et al. 1995 [52]	Y	Y	N	N	N	Y	?	N	N	?	?	High

Y = yes (green shading);? = unclear (amber shading); N = no (red shading).

1. Focused issue; 2. Appropriate methodology; 3. Case recruitment; 4. Control recruitment; 5. Exposure accurately measured; 6. Confounders identified and included in analysis; 7. Treatment effect; 8. Statistical precision; 9. Believability of results; 10. Generalisability; 11. Results consistent with other evidence.

### Personal factors

Gender as a predictor for hospitalisation due to LBP revealed inconsistent findings. Three studies found females were more likely to be hospitalised compared to males, including: a cross-sectional study of moderate risk of bias (PR 1.8, 95% CI 1.2–2.75) [28], a cohort study set in an emergency department with low risk of bias (OR 1.4, 95% CI 1.15–1.85) [49], and a cohort study set in a workplace of moderate risk of bias where the risk of hospitalisation for men was reduced (HR 0.46, 95% CI 0.26–0.79) [39]. One cohort study found men to have a greater risk of hospitalisation, but the population was significantly younger compared to the other studies examining gender (HR 3.2, 95% CI 2.7–3.7) [46]. There was no difference in hospitalisations between genders in four other cohort studies [27, 33, 44, 47].

Older age was a predictor of hospitalisation for LBP in people accessing a health service in five studies [27–29, 34, 47]. One cohort study with low risk of bias set in emergency departments found people 65 years of age and older had greater odds of hospitalisation compared to people of younger age (OR 3.0, 95% CI 2.59–3.59) [34]; and in a similar study also set in emergency departments, the odds were even greater in people over 85 years of age (OR 5.45 95% CI 5.34 5.57) [27]. Three other studies with moderate risk of bias found that the odds of hospitalisation increased for every year of age [29, 44] and decade of age [28, 47]. On the contrary, one cohort study with moderate risk of bias set in workplaces found workers between 30–39 years of age were at greater risk of hospitalisation due to LBP compared to older workers (RR 1.87, 95% CI 1.3–1.86) [53]; and a further two studies set in workplaces found the risk of hospitalisation for sciatica decreased with age (HR 0.59, 95% CI 0.38–0.9 women, HR 0.6, 95% CI 0.4–0.91 men) [32] and (HR 0.23, 95% CI 0.12–0.46) [33]. An experienced, work-hardened and physically fit workforce were explanations for reduced risk of hospitalisation in the older population. Two studies found no association between age and hospitalisation: one was under powered [51] and the other a retrospective cohort with high risk of bias [52]. There is moderate evidence that older age is associated with increased risk of hospitalisation for LBP in the emergency department context; while older age is associated with reduced risk of hospitalisation for LBP within workplaces.

Height, weight and body mass index were not predictors of hospitalisation due to LBP. One cohort study of moderate risk of bias conducted on a construction site found that men had greater risk of hospitalisation for LBP when body height was greater than 190 centimetres (RR 1.55, 95% CI 1.3–1.86) and body weight greater than 100 kilograms (RR 1.4, 95% CI 1.12–1.76) [53], but these results were not found by other studies [32, 33, 50, 52]. One cohort study examined childhood obesity but found no predictor of hospitalisation for LBP in adulthood [48].

Only one study examined ethnicity as a predictor of hospitalisation in LBP. The cohort study with low risk of bias set across North Carolina state health services found hospitalisations for LBP were decreased in non-white populations [37]. Whilst the reasons are unclear, racial barriers to access medical inpatient care were hypothesised by the authors.

### Health and lifestyle factors

General health status self-rated as poor increased the risk of hospitalisation due to sciatica in women (HR 1.75, 95% CI 1.02–2.98) and men (HR 2.0, 95% CI 1.32–3.03) in a large cohort study with moderate risk of bias set in a community clinic [32]. Weekly childhood health complaints were also associated with increased risk of hospitalisation for LBP in adulthood (HR 1.5, 95% CI 1.2–1.9) [46]. A further two studies found no association between self-reported health status and hospitalisation for LBP [28, 33]. There was also no association between disability status and hospitalisation for LBP (p>0.05) [37].

Comorbidities as predictors of hospitalisation for LBP were examined in a large cohort study set across inpatient and outpatient settings [47]. Comorbidities including anxiety, asthma, chronic obstructive pulmonary disease, depression, diabetes, gastrointestinal disorders, heart disease, hypertension, psychosis and rheumatoid arthritis were not associated with increased risk of hospitalisation. This study did find that people using opioids (OR 1.27, 95% CI 1.02–1.59) or non-steroidal anti-inflammatory medications (OR 1.76, 95% CI 1.43–2.17) prior to the initial episode of LBP had an increased odds of hospitalisation [47]. In a retrospective cohort study of high risk of bias, opioids also increased the odds of hospitalisations for LBP in people over 65 years of age with chronic LBP who initiated treatment with opioid therapies compared to people who initiated treatment with chiropractic spinal manipulations (OR 3.64, 95% CI 2.92–4.53) [55]. Frequent use of analgesia for all causes was similarly associated with increased risk of hospitalisation in a cohort study (RR 2.1, 95% CI 1.4–3.2) [36] but the risk of bias was high. Use of sedative medications prior to an initial episode of LBP did not increase the odds of hospitalisation in a cohort with moderate risk of bias [50].

Sleep disturbance as self-reported by participants in a cohort set in a workplace found increased risk of hospitalisation for LBP (RR 2.4, 95% CI 1.2–4.6) [43]. This finding was not found in a retrospective cohort of former athletes [52] or in a cross-sectional study [28]. The studies’ risks of bias, different populations and the imprecise measurement of sleep disturbance account for the inconsistency in results.

Smoking as a predictor of hospitalisation due to LBP revealed inconsistent findings. Three cohort studies with moderate risk of bias found current smokers were more likely to be hospitalised compared to non-smokers [32, 41, 53]. Two cohort studies of moderate to high risk of bias found that childhood smoking increased the risk of hospitalisation for LBP in adulthood [46, 48], the latter in males only. The other five studies found no risk of hospitalisation [28, 33, 36, 50, 52]. The inconsistency in results can be attributed to studies’ risk of bias and the lack of detail in defining the quantity of smoking.

Alcohol consumption as a predictor of hospitalisation for LBP also revealed inconsistent findings. Two cohort studies with moderate to high risk of bias found no association of consuming three to five drinks per day with risk of hospitalisation for LBP [50, 52]; while a cross-section study with moderate risk of bias found no alcohol consumption reduced the prevalence of hospitalisation for LBP (PR 0.58, 95% CI 0.37–0.91) [28].

Physical fitness and activity during leisure time were examined by eight studies [32, 33, 36, 38, 40, 48, 50, 52]. Whilst there was a trend in reduced risk of hospitalisation for LBP, the finding was only significant in one cohort study with high risk of bias (HR 0.40, 95% CI 0.21–0.79) [40]. Participants consisted of industrial workers who participated in strenuous physical activity during leisure time estimated at greater than 500 kilocalories/hour over three days a week. On the other hand, physical fitness measured using estimated maximal oxygen consumption (VO_2_ max) in workers in a prospective cohort study with moderate risk of bias was not associated with risk of hospitalisation for LBP (HR 0.88, 95% CI 0.51–1.50) [38]. One study examined childhood sports participation, but this too did not have predictive value on the risk of hospitalisation for LBP in adulthood [48]. Measurements of physical activity differed across studies and was imprecise creating inconsistencies in findings.

### Psychological factors

General stress and depressive symptoms were not associated with increased risk of hospitalisation for LBP [28, 42, 50]. There may be a risk for women (RR 2.1, p<0.001) as per one cohort study, but the result’s statistical precision is not reported and the risk of bias high [36]. In addition, a historical cohort study with high risk of bias examined personality traits, including extroversion, neuroticism and hostility as well as life satisfaction but found no increased odds of hospitalisation for LBP [52].

Beliefs about LBP were examined by two studies of moderate risk of bias. In a cross-sectional study of people with LBP in rural Ethiopia, hospitalisation for LBP was not linked to negative pain beliefs, such as perceived pain prognosis and life impact due to pain [28]. Odds of hospitalisation were increased in people presenting to an emergency department in Canada with LBP when they perceived the urgency of the emergency department visit as high, impact of pain on daily activities as severe, and expectation of being hospitalised [44]. Negative beliefs about LBP and treatment expectations may influence the risk of hospitalisation in emergency department settings.

### Socioeconomic factors

Living at home alone was associated with increased prevalence of hospitalisation for LBP (PR 2.54, 95% CI 1.34–4.15) in a cross-sectional study conducted in rural Ethiopia [28]. This study also found a lower prevalence of hospitalisation in rural areas (PR 0.55, 95% CI 0.34–0.9) attributed to socioeconomic and cultural factors in addition to health service access rather than pain burden [28]. The finding is in contrast to a retrospective cohort study with low risk of bias examining geographical variations across North Carolina state-wide health services where hospitalisations decreased in high urban populations [37]. A third cohort study of moderate risk of bias set in emergency departments in New South Wales, Australia found mixed results with odds of hospitalisation for LBP being greater for people residing in metropolitan areas (OR 1.36, 95% CI 1.27–1.46) followed by outer regional areas (OR 1.23, 95% CI 1.12–1.35) with the odds compared to inner regional areas, which had the lowest hospitalisation rates [30]. Whilst there are inconsistent findings relating to regional and metropolitan locations on the risk of hospitalisation for LBP, contextual factors unique to a geographical location may account for the variations observed in hospitalisation for LBP.

Compensable status of patients attending an emergency department with LBP may influence the risk of hospitalisation as per a retrospective cohort study with low risk of bias [27]. Patients insured through workers’ compensation schemes (OR 0.42, 95% CI 0.40–0.43), motor vehicle accident schemes (OR 0.90, 95% CI 0.86–0.94), and or overseas visitors (OR 0.74, 95% CI 0.68–0.81) had reduced odds of hospitalisations for LBP compared to publicly funded patients, while the odds increased in defence force personnel (OR 1.29, 95% CI 1.04–1.59) [27].

There were inconsistent findings in other socioeconomic factors. Identifying with middle social class in Finland increased the risk of hospitalisation for LBP compared to upper class (RR 4.0, 95% CI 2.3–6.8) in a cohort study set in community clinics [36], however this finding was not found in a cohort of male workers [50] in Denmark. Both studies had moderate to high risk of bias, studied different populations, and definitions of social class were imprecise likely impacting the consistency of results. High socioeconomic index was associated with higher odds of hospitalisation for LBP in a state-wide cohort study of low risk of bias set across emergency departments in New South Wales (OR 1.26, 95% CI 1.24–1.29) [27]. Lower education levels were associated with increased risk of hospitalisation for LBP in two cohort studies [45, 46] while no risk was observed in four other studies [28, 33, 37, 52]. The moderate to high risk of bias across studies and the different methods for defining low education similarly impacted the consistency of results. There were inconsistent finds on hospitalisation for LBP on employment status [37, 52]. There was no predictive value in marital status [36, 52] or household income [37, 45].

### Occupational factors

Occupation type as a risk of hospitalisation for LBP was examined by six studies [32, 36, 37, 39, 45, 53]. Blue-collar workers had increased risks compared to white-collar workers in five of the studies, all of which had moderate to high risk of bias. Occupations reported as having increased risks included manual labourers [39, 45, 53], metal and machine workers [36, 53], industrial workers [36, 53], and nurses [32]. There was inconsistent evidence amongst studies for workers in building trades [32, 53], agriculture and forestry [32, 36], sales [36] and transport [32, 37, 53]. Most of the studies were biased by excluding women. Only one study equally represented both genders and concluded that the risk for manual labourers was present in both men and women [45].

Physical demands of work were examined by five cohort studies with moderate to high risk of bias [33, 42, 50, 52, 54]. Sedentary workers who performed occasional heavy duties had an increased risk of hospitalisations in two studies [33, 50]. The evidence for hospitalisation in workers who perform regular heavy duties was inconsistent, where one study found performing regular heavy duties was protective and may reflect workers’ physical fitness (HR 0.48, 95% CI 0.26–0.89) [33] while another study found heavy duties increased the risk of hospitalisation (HR 2.37, 95% CI 1.36–4.13) [50]. Specific work tasks, including lifting was associated with hospitalisation [33], but awkward postures, prolonged sitting, constant and repetitive movements, or task variety and flexibility did not change the risk [33, 42, 52]. Exposure to vibrations increased the risk of hospitalisation due to LBP in a large cohort of male workers with low risk of bias (OR 1.35, 95% CI 1.12–1.63) [54] but this was not found in a much smaller cohort with sciatica with moderate risk of bias [33].

Work-absenteeism due to LBP was associated with increased risk of hospitalisation for LBP (RR 3.3, 95% CI 1.61.67) [39] as was low supervisor support (RR 2.9, 95% CI 1.3–6.3) [42] in cohort studies. There was no predictive value in mental stress at work [50] or co-worker support [42].

### Clinical assessment findings

Pain intensity was associated with increased hospitalisation for LBP when presenting to an emergency department with the odds increasing for each additional point on a 11 point numerical rating scale (OR 1.95, 95% CI 1.14–3.33) in a cohort study with moderate risk of bias [51]. Hospitalisation was also more prevalent amongst community dwellers in a cross-sectional study who rated their pain intensity as moderate (PR 2.46, 95% CI 1.15–5.52) and severe (PR 8.84, CI 4.82–18.13) [28]. The risk of hospitalisation was also increased for pain distress symptoms (HR 3.69, 95% CI 1.51–9.05) [39] and past history of low back pain [39, 47] in cohort studies with moderate risk of bias.

There were inconsistent findings amongst studies on the predictive value for hospitalisations for LBP when the symptoms included leg pain [28, 39], physical examination findings including neurological deficits [39, 51] and presence of serious spinal pathology or red flags [34, 51]. There was no evidence of increased risk of hospitalisation for people with previous attendance to an emergency department with LBP [51] or with duration of current episode of symptoms [51].

### Health systems and processes

When attending an emergency department with LBP, the risk of hospitalisation is increased when a person arrives via an ambulance compared to self-presentation. The odds were lower in a private hospital (OR 2.03, 95% CI 1.06–3.90) where patients’ average age was older and hospitalisation rates significantly higher compared to other studies [29], while the odds were higher in a prospective cohort of patients participating in a survey (OR 4.95, 95% CI 1.79–13.7) [44]. Attending an emergency department during working hours (OR 1.74, 95% CI 1.48–2.05), and being triaged as urgent (OR 3.37, 95% CI 1.48–9.38) or semi-urgent (OR 2.99, 95% CI 1.37–6.48) [34] increased the odds of hospitalisation; as was being referred to an emergency department by a private doctor (OR 2.18, 95% CI 2.06–2.30) [30]. There was no difference in hospitalisation rates when the treating clinician was an emergency specialist physician compared to a visiting medical officer specialist [30]. Patients treated by a medical officer or nurse practitioner in the emergency department had greater odds of hospitalisations for LBP (OR 3.38, 95% CI 2.33–4.91) compared to being treated by an advanced practice physiotherapist [49], likely attributed to physiotherapists managing cases of lower urgency. These studies were of low to moderate risk of bias and highly applicable to the Australian healthcare context.

Healthcare access and resource utilisation in emergency departments also predict hospitalisations for LBP. In a private emergency department set in a high socioeconomic area, the odds of hospitalisation for LBP increased when ordering pathology tests (OR 3.32, 95% CI 2.01–5.49), ordering computed tomography images of the lumbar spine (OR 1.86, 95% CI 1.12–3.11) and with longer treatment times in emergency departments (OR 1.16, 95% CI 1.07–1.26) [29]. In a cohort study with low risk of bias set across a state-wide health service, hospitalisation rates for LBP increased at hospitals with computed tomography or magnetic resonance imaging availability [37]. However, hospitalisations did not increase when there was high hospital unoccupied bed density (p<0.05) suggesting bed availability does not drive hospital utilisation for LBP [37].

Hospital contextual factors also accounted for hospitalisations for LBP. A retrospective study of moderate risk of bias audited 37 emergency departments in New South Wales and found that hospitalisation rates for LBP were greater amongst centres with higher specialty classifications (levels 1–3) compared to lower speciality classifications (levels 4–6) (OR 1.22, 95% CI 1.14–1.30) [30]. A state-wide audit of emergency departments across New South Wales with low risk of bias estimated that hospital factors (geographical location, hospital specialty classification) explained 10% of variations in hospitalisations for LBP (ICC = 0.10) whilst controlling for patient and clinical factors; with a median odd ratio of 2.03 at hospitals with higher hospitalisation rates [35].

There was no association between hospitalisation for LBP and access to primary care physicians, spinal surgeons or chiropractors (p>0.05) [37]. On the other hand, when receiving physical therapy interventions in primary care for any condition, the risk of hospitalisation for LBP was reduced for up to 180-days of receiving physical therapy (RR 0.68, 95% CI 0.64–0.72) [31]. This cohort study had a high risk of bias with inadequate descriptions of interventions to reproduce results and unknown effect sizes to demonstrate clinical impact.

### Summary of results

There is moderate level evidence that arrival to an emergency department via ambulance with LBP, and older age increase the risk of hospitalisations for LBP. There is low level evidence that high pain intensity, past history of LBP, opioid use, and occupation type predict hospitalisation for LBP. There is emerging evidence from single study findings of increased risk of hospitalisation for LBP associated with pain distress, residing alone, ethnicity, compensable status, younger workers, residential and hospital contextual factors, emergency department triage category, hospital specialty classification, and utilisation of emergency department investigational resources. There was no evidence for gender, height and weight, self-rated health status, comorbidities, stress and depression symptoms, smoking, alcohol consumption, socioeconomic status, education level, employment status, duration of pain, leg pain, clinical examination findings, or primary care access on the risk of hospitalisation for LBP.

### Grade of body of evidence

The FORM framework [25] was used in the synthesis process and the findings are described in Table 4. The body of evidence was graded as satisfactory with support for some of the research findings, but caution advised due to methodological concerns, including inconsistencies in outcome measurements, lowering the grade of the body of evidence. The clinical impact and generalisability of the evidence found in this systematic review will most likely benefit people presenting to an emergency department with LBP.

**Table 4:** FORM framework analysis of the grade of body of evidence [25].

Component	Grade	Comments
Evidence base	B–Good One or two level II studies with a low risk of bias, or a systematic review or several level III studies with a low risk of bias.	*Quantity of evidence*: 23 studies across 29 articles with 2,462,497 participants. *Levels of evidence & risk of bias*: Level II: 12 articles (1 low risk of bias, 6 moderate risk of bias, 5 high risk of bias). Level III: 16 articles (5 low risk of bias, 6 moderate risk of bias, 5 high risk of bias). Level IV: 1 article (moderate risk of bias).
Consistency	C–Satisfactory Some inconsistency reflecting genuine uncertainty around clinical question.	Statistical significance reported in 80% of articles. Inconsistent findings found in 25% of variables predicting hospitalisation for LBP. Study designs mostly cohort studies. Variable clinical contexts. Variable outcome measures and timepoints when outcomes were measured.
Clinical impact	C–Satisfactory Moderate clinical impact.	Clinical impact discussed in 60% articles. Key factors identified with risk of hospitalisation for LBP are strengthened by relevance to the clinical contexts, statistical precision of confidence intervals, and clinical importance of the findings.
Generalisability	B–Good Populations studied in the body of evidence are similar to the targeted population.	Studies set in 8 different countries, though only 1 developing country. Different clinical contexts. Generalisability of findings was poorly discussed by studies.
Grade of body of evidence	C–Satisfactory Body of evidence supports some of the research findings but care should be taken in its application.	Most studies were found to be of low to moderate methodological quality. Consistency of findings was impacted by the heterogeneity of outcome measurement. Clinical impact and generalisability of the evidence base will benefit targeted populations within specific contexts, such as emergency departments.

## Discussion

While hospital-based care is sought for management by many people experiencing LBP, to date there has been limited quality research evidence to determine the predictors of hospitalisation for LBP. This systematic review aimed to address this knowledge gap and identified a body of evidence consisting of 23 studies published over 29 articles and examining 52 predictor variables. The summarised findings indicate that there is moderate level evidence that arrival to emergency departments with LBP via ambulance and older age; and low level evidence that high pain intensity, past history of LBP, opioid use, and occupation type increase the risk of hospitalisation for LBP.

Closely related to age are other variables such as functional capacity, fitness and sedentary lifestyle [59, 60]. Inactivity may be an underlying risk factor of hospitalisation for LBP or sciatica as was found by studies of sedentary workers who occasionally performed heavy work duties [33, 50]. However, studies that directly measured physical activity time or fitness did not show a reduction in hospitalisation, probably from imprecise measures. The observation of reduced risk of hospitalisation for LBP or sciatica in older workers who are physically conditioned and experienced in performing their work duties [33, 53] likely indicates that functional capacity and fitness modify the effects of ageing, which is consistent with the literature [61–63], countering the risks of hospitalisation. The higher odds of hospitalisation for LBP in the cohort with younger workers also challenged the commonly held belief that ageing and lumbar disc disease are associated with LBP [53]. The poor correlation of spinal radiological findings with LBP has been well established [64].

There remains a large knowledge gap for predictors of hospitalisation for LBP. There were no identified studies examining functional impairment and very few examining psychological factors. This review found some pain behaviours to be factors in hospitalisations for LBP, such as prior analgesic use, pain distress, and attending an emergency department for LBP via an ambulance. Low pain self-efficacy may be a characteristic of people hospitalised with LBP, but this has not been investigated. Negative beliefs about pain were associated with hospitalisations within the context of emergency departments [44], particularly around illness perception and patients’ urgency for investigation and hospital-based care. Helplessness and expectations of medical interventions to allow return to premorbid function were common themes from a qualitative study of patients hospitalised for LBP [19]. Escalating pain levels, distress and disability attributed to personal and contextual factors influenced patients’ decisions to attend an emergency department for non-specific pain conditions [65–67]. While there is support for psychological factors as components of the pain experience in people with LBP in emergency departments or hospitals, this review did not find studies of specific psychological factors, such as catastrophizing, depression or anxiety, to predict hospitalisation for LBP.

Social and environmental contributors to hospitalisation for LBP are also suspected from this review such as geographical location, but specific causations are unknown. Residing in regional or metropolitan areas was not a consistent theme of hospitalisations for LBP in this review; rather, social and cultural influences of a local community and health system accounted for some of the geographical variations in hospitalisation for LBP. Clinician biases [35, 37], patient cultural expectations of hospital-based care [28, 44] and lack of outpatient social support [35] are hypothesised as contributors to hospitalisation for LBP. The literature highlights the broad influence of social factors on outcomes of people with LBP, such as residing in regional or rural areas [68], work and financial stressors [68, 69] and inadequate social supports [69, 70].

Understanding the social and contextual factors of LBP is highlighted by this review to better recognise risk factors for poor prognosis and avoid escalating medical care such as hospitalisation in people with LBP. Clinical practice in hospitals and emergency departments has a primary focus on biomedical factors given the priorities in managing serious pathology and acute and urgent care [71]. Operating purely in this framework for non-specific pain conditions, such as LBP and sciatica may contribute to escalation of low-valued care [6, 72] including over investigation and intervention [12], and increased health costs and inefficiencies [17]. Contextual factors can not be ignored in the management of musculoskeletal conditions if it is to be person-centred and high-valued care [73, 74], and this is ultimately best delivered in community health settings [75].

The quality and access to community-based care are important in improving outcomes of people with LBP [58, 76]. Poor access and availability to community medical and allied health services, health costs, and funding models are barriers to equitable care and may contribute to escalation of management of LBP [77]. This review failed to find sufficient studies exploring whether limited access to community-based care contributes to hospitalisations for LBP.

### Limitations

As with any research, there are limitations to this systematic review. While the systematic searching of the literature identified a large body of evidence to inform the review topic, there were concerns regarding the methodological quality of the included studies. An area of concern is the subjective and psychometrically untested measurements of predictor variables, such as occupation type, physical activity levels, stress levels and socioeconomic status, rendering the measures as imprecise and requiring caution with interpretation of the results. The long follow-up periods weakened the associations of certain baseline predictor variables with hospitalisation outcome at baseline, especially for variables whose conditions are not fixed and can change over time, such as smoking status or body weight. While this systematic review process was underpinned by best practice in the conduct of systematic reviews (PRISMA), likely publication and language bias should be acknowledged. While strategies were implemented to avoid publication bias, such as grey literature and secondary searching, due to the complexity and imprecise nature of searching, some publications may have been missed.

### Implications for practice

With the emerging evidence of various predictors of hospitalisation for LBP, there are implications for clinical practice in different settings. After exclusion of red flags and serious pathology, clinicians in emergency departments should screen patients’ risk of hospitalisation for LBP, such as age, pain intensity, distress and mode of arrival. Artificial intelligence may have a role in predictive modelling, patient monitoring and hospital operations and there has been evidence of its use in emergency departments for a variety of health conditions [78]. Resources can be better directed based on risk profile [79, 80], such that lower risk patients are provided with reassurance, simple analgesia, education on self-management with follow-up in community-based care; while higher risk patients may benefit from input by clinicians skilled in communicative and educational approaches, optimisation of conservative management including early mobilisation and physiotherapy, a stepped approach to pharmacological management of pain, and early referral to pain management services. These approaches are emerging as evidence-based practices in emergency departments [71, 81, 82] but the effect on hospitalisations continues to be challenging. Policy makers and hospital administrators should consider innovative, alternative pathways to emergency departments for people with LBP, such as virtual hospital care [83], especially for people arriving to emergency departments via ambulance. Hospital avoidance strategies will have the greatest impact by improving access to community-based care strengthened by partnerships with hospital health services [72, 74, 84, 85].

### Implications for research

Whilst this review found evidence of some predictors for hospitalisation for LBP, methodological concerns of the current evidence may guide the direction of future research. Future research should focus on psychological and social factors that may predict hospitalisation for LBP. Such factors are poorly documented in hospital medical records [86] and therefore reliance on clinical audits is limited and requires mixed-methods approaches [87], including qualitative research that explore the experiences of people with LBP and their navigation of health systems. Community healthcare access and utilisation also warrants further research on the risk of hospitalisation for LBP.

## Conclusions

This systematic review, first of its kind, demonstrates the complex and multifactorial contributors to hospitalisation for people with LBP with or without leg pain. Specifically, arrival at an emergency department via an ambulance and older age appear to increase the risk of hospitalisation within this cohort. These findings could inform healthcare stakeholders’ decision-making in implementing evidence-informed strategies to better manage the condition and more efficient resource allocation.

## Supporting information

S1 Checklist PRISMA checklist. (DOCX)

S1 Appendix Search strategy. (DOCX)
