# Supplementary material for: Systematic review of predictors of hospitalisation for non-specific low back pain with or without referred leg pain
Source: PLoS One. 2023 Oct 10;18(10):e0292648. doi: 10.1371/journal.pone.0292648 (PMC10564130; doi:10.1371/journal.pone.0292648)
Supplement: S1 Appendix — (DOCX) [file pone.0292648.s002.docx]

**S1 Appendix. Search Strategy.**

**Research Question:** What are the predictors of hospitalisation in adults with non-specific low back pain with or without referred leg pain?

**PICOTS**

| **Population** | Adults 18 years or older at the time of hospitalisation for non-specific low back pain with or without leg pain. |
| --- | --- |
| **Index prognostic factors** | Demographic, clinical findings, medical investigations, hospital operational processes, contextual factors, etc. |
| **Comparator** | Not applicable. |
| **Outcome** | Hospitalisation in a tertiary hospital due to non-specific low back pain. |
| **Time** | All time points including pre, during or post low back pain episode. |
| **Settings** | All clinical contexts will be considered, including community health clinics, emergency departments, inpatients or outpatients. |

**Search strategy:** Incorporated into electronic databases (Ovid Medline, Emcare, Embase, PsycINFO, Cochrane Library, PEDro and OTSeeker).

| Search no. | Search terms |
| --- | --- |
| #1 | exp back pain/ |
| #2 | exp low back pain/ |
| #3 | exp backache/ |
| #4 | exp sciatica/ |
| #5 | exp hospitali$ation/ |
| #6 | 1 or 2 or 3 or 4 |
| #7 | 5 and 6 |

Limits: English language

Date: Inception to 5^th^ Oct 2022
